# Computational modelling suggests that Barrett’s oesophagus may be the precursor of all oesophageal adenocarcinomas

**DOI:** 10.1136/gutjnl-2020-321598

**Published:** 2020-11-24

**Authors:** Kit Curtius, Joel H Rubenstein, Amitabh Chak, John M Inadomi

**Affiliations:** 1 Centre for Genomics and Computational Biology, Barts Cancer Institute, School of Medicine and Dentistry, Queen Mary University of London, London, UK; 2 Division of Biomedical Informatics, Department of Medicine, University of California San Diego, La Jolla, California, USA; 3 Division of Gastroenterology, University of Michigan, Ann Arbor, Michigan, USA; 4 Center for Clinical Management Research, Ann Arbor Veterans Affairs Medical Center, Ann Arbor, Michigan, USA; 5 Division of Gastroenterology and Liver Disease, University Hospitals Cleveland Medical Center, Case Western Reserve University School of Medicine, Cleveland, Ohio, USA; 6 Department of Medicine, University of Utah School of Medicine, Salt Lake City, Utah, USA

**Keywords:** Barrett's oesophagus, Barrett's carcinoma, oesophageal cancer, screening, pre-malignancy - gi tract

## Abstract

**Objective:**

Barrett’s oesophagus (BE) is a known precursor to oesophageal adenocarcinoma (OAC) but current clinical data have not been consolidated to address whether BE is the origin of all incident OAC, which would reinforce evidence for BE screening efforts. We aimed to answer whether all expected prevalent BE, diagnosed and undiagnosed, could account for all incident OACs in the US cancer registry data.

**Design:**

We used a multiscale computational model of OAC that includes the evolutionary process from normal oesophagus through BE in individuals from the US population. The model was previously calibrated to fit Surveillance, Epidemiology and End Results cancer incidence curves. Here, we also utilised age-specific and sex-specific US census data for numbers at-risk. The primary outcome for model validation was the expected number of OAC cases for a given calendar year. Secondary outcomes included the comparisons of resulting model-predicted prevalence of BE and BE-to-OAC progression to the observed prevalence and progression rates.

**Results:**

The model estimated the total number of OAC cases from BE in 2010 was 9970 (95% CI: 9140 to 11 980), which recapitulates nearly all OAC cases from population data. The model simultaneously predicted 8%–9% BE prevalence in high-risk males age 45–55, and 0.1%–0.2% non-dysplastic BE-to-OAC annual progression in males, consistent with clinical studies.

**Conclusion:**

There are likely few additional OAC cases arising in the US population outside those expected from individuals with BE. Effective screening of high-risk patients could capture the majority of population destined for OAC progression and potentially decrease mortality through early detection and curative removal of small (pre)cancers during surveillance.

**Significance of this study**

**What is already known on this subject?**

- Barrett’s oesophagus (BE) patients have a 40–50-fold higher risk of developing oesophageal adenocarcinoma (OAC) than the general population yet many remain undiagnosed.
- Identified BE patients receiving surveillance can have early cancers discovered endoscopically, which decreases the high overall OAC-associated mortality.
- Currently, around 90% of patients who develop OAC were never part of a BE surveillance programme, and those BE patients on surveillance have a low annual progression rate of 0.1%–0.3% to develop OAC.

**What are the new findings?**

- By applying a model that incorporates the evolution from normal cells to BE to OAC in patients, we found that the numbers add up—the expected number of OAC cases in the US population are explained by the published rates of BE described above.
- We cohesively examined the published estimates to determine that all OAC likely arises from both identified BE and occult, undiagnosed BE in the population.

**How might it impact on clinical practice in the foreseeable future?**

- Based on current best estimates, our findings suggest that there is no public health need to seek cases of a non-BE alternative pathway to OAC.
- Increasing efforts for effective, sensitive screening and surveillance of the true BE population has the potential to decrease OAC mortality in the coming years.

## Introduction

Oesophageal adenocarcinoma (OAC) is typically diagnosed when a patient presents with symptoms such as dysphagia. Unfortunately, the majority of these patients do not live past the first year of their diagnosis because by the time dysphagia develops, metastatic cancer is already present. In order to prevent this cancer or detect it at an earlier, more treatable stage, efforts are now made to identify patients with Barrett’s oesophagus (BE), the only known precursor to OAC. Identified BE patients are believed to have a 40–50-fold higher annual incidence of OAC than the general population.^1^ Metaplastic BE progresses through dysplasia to cancer. Advances in endoscopic eradication therapy for dysplastic BE discovered during surveillance of BE can now prevent cancer.^2^ However, most cancers arise in patients without previously diagnosed BE suggesting either inadequate screening strategies or, as a recent study proposes, the possible existence of a pathway independent from the BE pathway.^3^ In this study, we seek to answer a simple question about the unseen origins of OAC: does overall OAC incidence reflect the number of cancers that would be expected to arise only from prevalent BE? In other words, do any OAC cases remain unaccounted for that ergo did not arise from the typical Barrett’s precursor pathway? The answer to this question will importantly guide research and public health efforts. If BE is the major or only precursor of OAC, then investigators should continue to focus on improving BE detection. If BE is not the major precursor of OAC, then research needs to focus on identifying alternative pathways and BE screening programmes will have limited impact on prevention and early detection of OAC.

In reality, very few individuals who have BE are ever offered an upper endoscopy, and therefore most BE remains asymptomatic and undiagnosed.^1^ Patients with gastro-oesophageal reflux disease (GERD) are technically the only subpopulation of the general public typically recommended BE screening because it is believed they have a 5-fold relative risk (RR) of developing long segment BE,^4^ yet even so only about 10% of GERD patients will receive an endoscopy.^1^ This indicates *underscreening*, likely because patients either do not complain of their GERD symptoms, they respond adequately to medical therapy, or were otherwise not deemed suitably high-risk by their physician to warrant an esophagogastroduodenoscopy. Nonetheless, the prevalence of BE in the general population is 1%–2%, whether diagnosed or not,^5 6^ and this is likely considerably higher in certain at-risk groups in the USA.^7–10^ The main concern is that the average rate to develop OAC in these patients is low—around 0.3% per year.^11^ Therefore, the majority of endoscopies are futile in finding OAC. We aimed to answer whether all prevalent BE expected, diagnosed and undiagnosed in the US population, could account for all the incident OACs expected as progression rates would imply, to fit the national cancer registry data.

## Methods

The question above is too complex to answer on the ‘back of an envelope’ because published *average* rates of progression are dependent on age, birth cohort and calendar year. In particular for OAC, age-specific incidence rates vary drastically between men and women.^12^ This complexity of timescales involved in normal to premalignant BE to OAC progression has necessitated the creation of quantitative models that analyse cancer incidence rates, and project these trends into the future for public health risk assessments and planning.^13^ Models also quantify the potential impact of progression rates measured in clinical studies on hypothetical intervention and surveillance scheduling in efficacy and cost-effectiveness studies.^14–16^ Such models allow us to perform quantitative, comparative analyses on the benefits versus harms of proposed screening and surveillance protocols against watch-and-wait strategies; these simply cannot be done heuristically due to the complex nature of cancer evolution.

**Figure 1:**
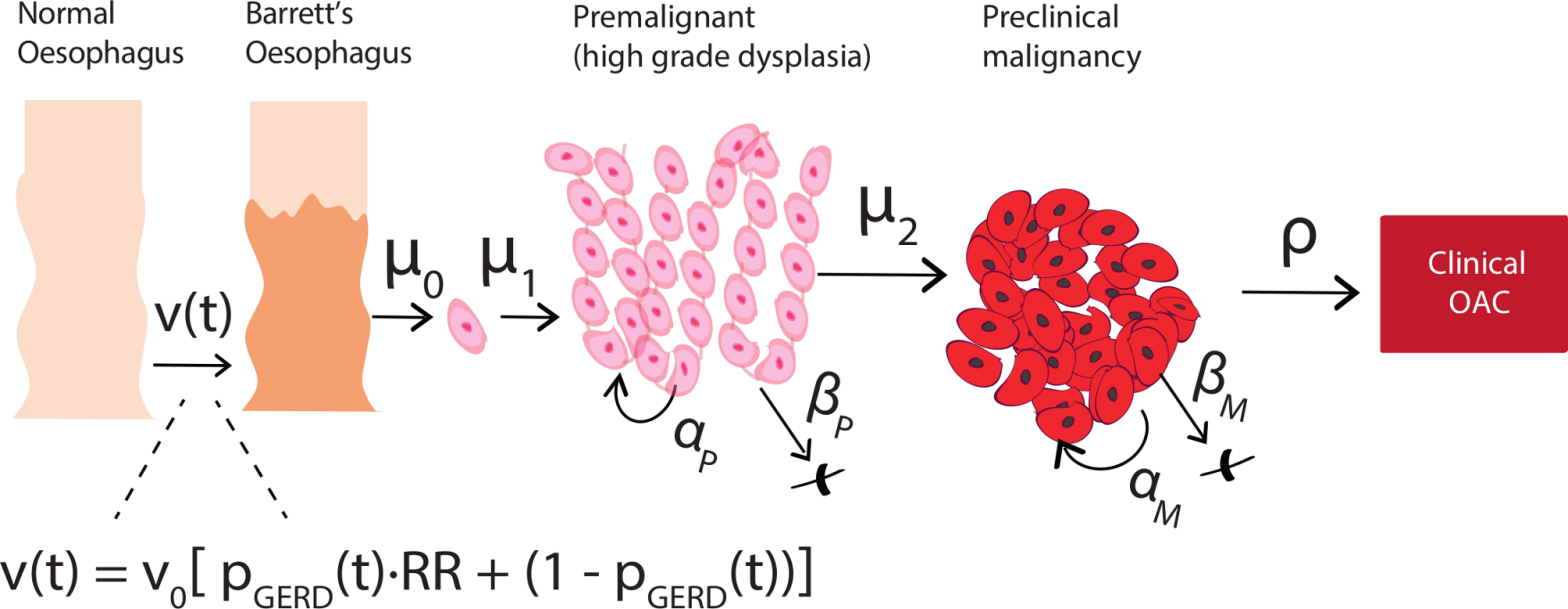
The stochastic, multiscale model for OAC development (MSCE-OAC) includes conversion from normal squamous epithelium in the oesophagus to BE metaplasia with BE onset rate ν(t), which is a function of a baseline rate ν_0_ and age-dependent prevalence of GERD p_GERD_(t) (see Methods for details). Two-hit processes with rates μ_0_, μ_1_ can initiate a premalignancy (eg, inactivation of tumour suppressor gene *TP53* in non-dysplastic BE due to mutation/copy number alteration in a BE daughter cell creates first cell of a high grade dysplasia lesion). Premalignant cell growth rates are defined as α_P_ = division rate, β_P_ = death/differentiation rate per year. Malignant transformation with rate μ_2_ creates the first cell of a preclinical clone that can grow with rates α_M_ = division rate, β_M_ = death/differentiation rate per year. Size-based probability ρ for detection of preclinical malignant clone can lead to patient-specific time of incident OAC. BE, Barrett’s oesophagus; OAC, oesophageal adenocarcinoma; GERD, gastro-oesophageal reflux disease; MSCE-OAC, multistage clonal expansion for oesophageal adenocarcinoma.

**Figure 2:**
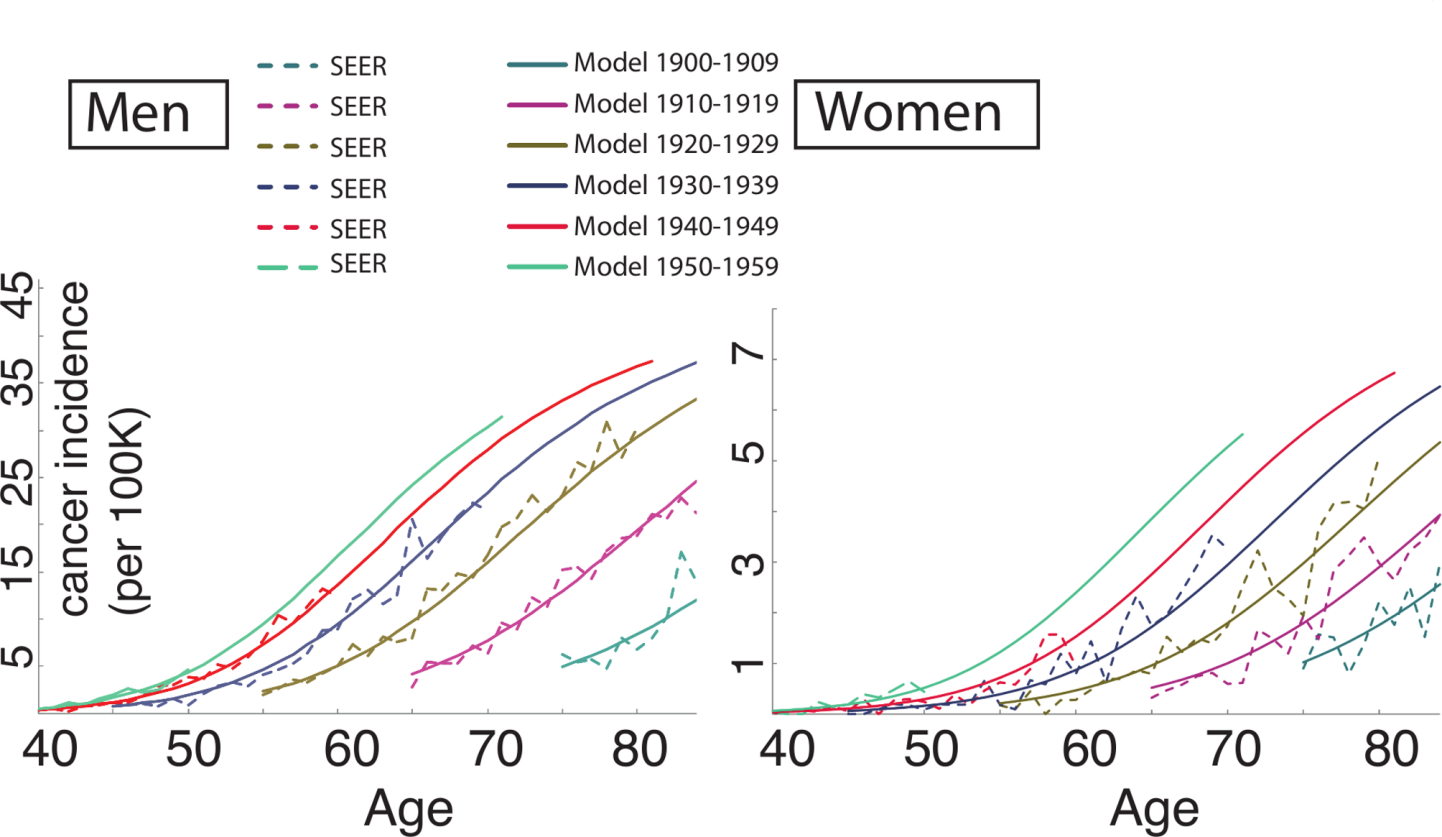
The MSCE-OAC model was previously calibrated to SEER incidence curve data stratified by sex and 10-year grouped birth cohorts from 1900 - 1909 to 1950–1959.^13^ The model hazard fits by birth cohort (denoted by colour) represent OAC incidence curves (solid lines) that are consistent with Surveillance, Epidemiology and End Results (SEER) data trends by birth cohort (dashed lines), separately for men (left panel) and women (right panel). MSCE-OAC, multistage clonal expansion for oesophageal adenocarcinoma.

In this study, we model both the onset of BE and the progression of BE to OAC. As a brief background, the multistage clonal expansion model for OAC (herein referred to as the *MSCE-OAC* model, but also referred to as the *MSCE-EAC* model elsewhere) is a stochastic model for development of OAC during patient lifetime that includes probabilities of developing BE at various ages, followed by initiation of dysplastic and malignant cell clones in BE with parameters for growth and progression of individual clones to cancer (figure 1). The *inputs* only include GERD prevalence (calibrated to age-specific and sex-specific estimates)^17 18^ and OAC age-specific and sex-specific incidence curves provided by Surveillance, Epidemiology and End Results (SEER) registry.^12^ The BE prevalence and neoplastic progression rates are calibrated to fit those inputs, that is, they are not based on observed BE prevalence nor neoplastic progression rates from empiric studies. Briefly, the model includes a GERD-stratified risk curve to develop BE, which is modelled as an age-dependent rate of exponential BE onset each calendar year with an unknown baseline parameter ν_0_. The patient-specific BE lengths can vary, derived from a Beta distribution with general population mean length set to 2–3 cm. Beyond ν_0_, the baseline constant rate for BE onset, the additional model parameters govern the evolutionary dynamics for dysplastic and malignant growth and OAC detection. The model parameters have been previously calibrated such that the resulting hazard functions fit to OAC age-specific and sex-specific incidence curves provided by SEER registry.^13^ We found during rigorous model selection with likelihood ratio tests that models stratified by birth cohort and sex best fit the incidence data, robust to sensitivity analyses (figure 2). With these fits, the model *outputs* used for this study include the expected number of OAC cases in an at-risk population at a given year calculated using the hazard function *h*_OAC_ (see online supplemental material for equation details), along with the BE prevalence and the resulting BE-to-OAC progression rates (predicted as specific to age, sex and birth cohort).

10.1136/gutjnl-2020-321598.supp1 Supplementary data

This model has been used and improved in comparative analyses within the NCI Cancer Intervention and Surveillance Modelling Network consortium for the past 9 years, which has enabled numerous studies on sensitivity of biopsy sampling techniques for detection of small dysplastic lesions,^14^ on influence of patient-specific molecular BE dwell time on future OAC risk,^19^ and on cost-effectiveness of endoscopic eradication therapy for certain BE risk groups during surveillance.^15^ In our original study on modelling OAC incidence and mortality rates from 1975 to 2010, we used SEER-specific model fits combined with US census data to estimate past and predict future OAC-related deaths but did not include predicted OAC cases by calendar year when applied to US census data.^13^

In the Results below, we expand on prior modelling to help elucidate an answer to our general public health question—‘Is BE the precursor of all OAC?’ To do this, we first applied the model to estimate the number of OAC cases using the US age-specific and sex-specific at-risk population estimates from the US census data, to be able to compare with the expected number quoted by Vaughan and Fitzgerald.^1^ This outcome serves as an independent validation of successful calibration of our model to OAC incidence. Then, we compared the simultaneous predictions of age-specific BE prevalence using the MSCE-OAC model with the published data currently used for screening rationale,^20^ which included endoscopic reports from the Clinical Outcomes Research Initiative (CORI) for more than 150 000 patients, most of whom were born around 1950. We also compared the mathematical predictions of neoplastic progression rate from non-dysplastic BE to published estimates.

## Results

First, Vaughan and Fitzgerald estimated that the newly diagnosed number of cases for ages greater than 40 to be roughly around 10 000 total in the USA every year based on data from 2010 with an average OAC incidence rate across all age groups.^1^ With the Markov model framework, we can analytically compute the OAC hazard function and estimate the expected number of newly diagnosed OAC cases by age and year separately for men and women when considering also population data. As a starting point using 2010 census person-year data,^21^ the model predicts that about 2.2 million adults had prevalent BE in 2010, which is around 1.6% of the general US population over age 40. Then, for age groups greater than 40 in both sexes of all races, our single-age calibrated model estimated that the expected number of new OAC cases diagnosed in 2010 was equal to 9970 (95% CI: 9140 to 11 980).

We also computed the analogous estimate for OAC cases using incidence rates quoted directly from the SEER registry for ages 40–90, which was found to be 9400 OAC cases total in 2010.^12^ Thus, the estimate generated by our computational model of progression from BE to OAC is closely consistent with the total number of OAC cases reported in SEER, which also aligns with the 10K incident cases quoted by Vaughn and Fitzgerald.^1^ The model therefore suggests that over 90% of OAC cases are attributable to BE.

**Figure 3:**
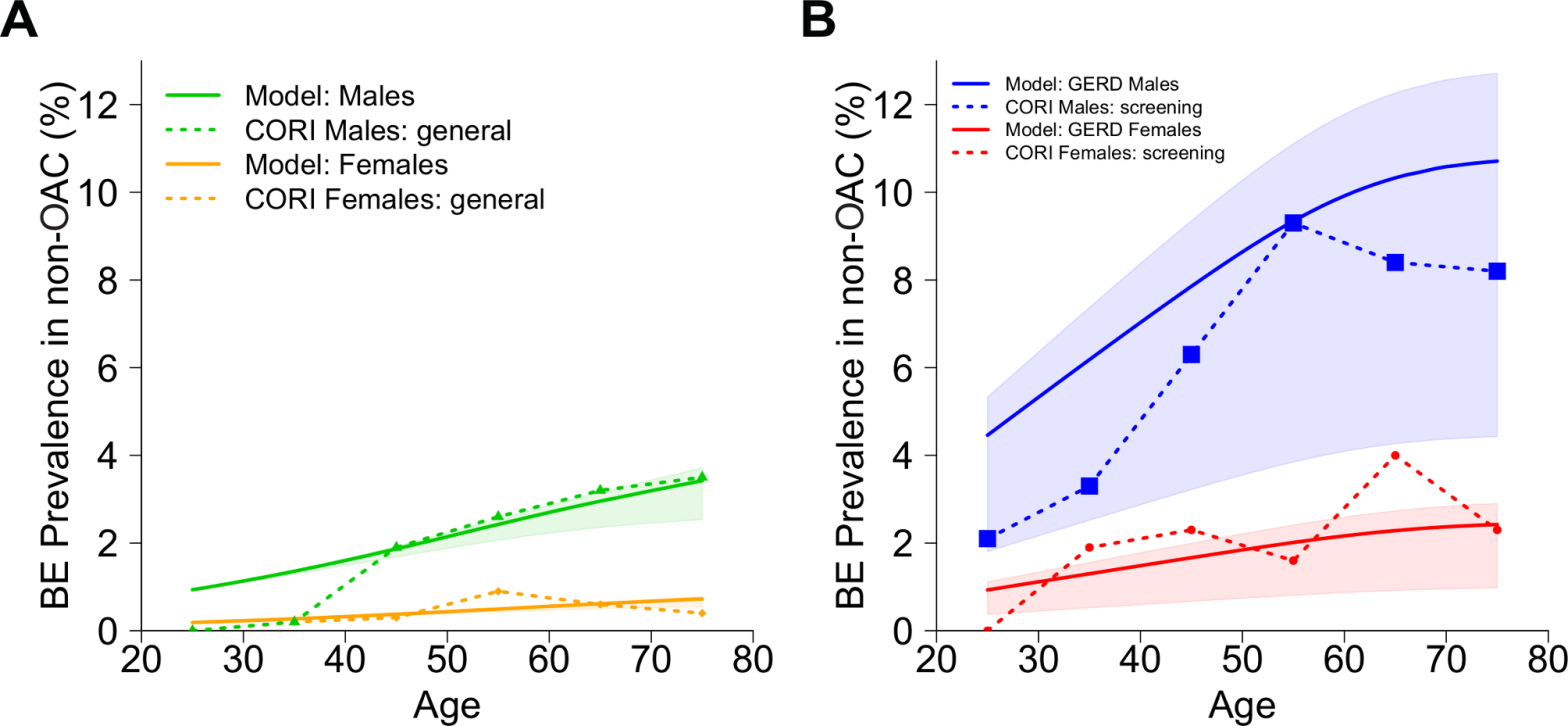
Model predictions for BE-positive yield in a cancer-free population (solid lines) are consistent with observed data (dashed lines) from Clinical Outcomes Research Initiative (CORI).^20^ (A) Solid lines show model results for the general US population stratified by sex from the 1950 birth cohort, with contributions of relative risk (RR) of BE from the age-specific, prevalent GERD population assumed to be RR=5 (shaded areas, RR=[2,6]). Dashed lines show consistency with observed BE prevalence data for patients without indication for screening in CORI, which are independent of the model. Model BE prevalence estimates are part of the evolutionary multistage process and thus affect predictions of the total OAC cases predicted (see Results). (B) Solid lines show model results for the symptomatic GERD subpopulation stratified by sex from the 1950 birth cohort with RR for BE set to RR=5. The shaded areas are predicted ranges for GERD subpopulations with fixed RR=2–6 to describe a wide range of increased risks of BE in published estimates, based on factors such as onset age of GERD and BE length. The true GERD-specific BE prevalence contributing to mathematical formulation used in (A) is within this region, where individual contributions are based on GERD onset age and underlying distribution of RR. Dashed lines show BE prevalence data for patients with GERD, and/or another indication for screening, in CORI. BE, Barrett’s oesophagus; GERD, gastro-oesophageal reflux disease; OAC, oesophageal adenocarcinoma.

Second, we considered what the model simultaneously predicted for BE prevalence and BE-to-OAC progression rates in order to achieve the expected ~10K cases. Breaking down the contributions of the 2.2 million total BE patients estimated above, the model predicted BE prevalence to be 1.9%–2.4% in men and 0.4%–0.5% in women in the general US population ages 45–55 in 2010 (figure 3A). These predictions concur with best estimates^5 6^ and influence the total OAC cases predicted by the multistage model. To further explore implications for high-risk patients, we note that the model predicted a BE prevalence of 7.9%–9.3% in US men with symptomatic GERD who are cancer-free ages 45–55 in 2010 when the RR of BE vs non-GERD individuals is assumed to be RR=5 (figure 3B). This is also consistent with the estimate of 8% provided by Vaughan and Fitzgerald^1^ for prevalence of cancer-free BE diagnoses among GERD patients who undergo an upper endoscopy. Further, the model’s predicted age-specific BE prevalence curves by sex were consistent with previous results on BE prevalence from the CORI study^20^ (figure 3A). Compared with our model results and 8% quoted above^1^ for high-risk groups, the CORI study independently found similar BE prevalence in white men with GERD of 6.3% for ages 40–49 and 9.3% for ages 50–59 (figure 3B). To account for likely heterogenous RR of developing BE in GERD populations based on symptom onset age, BE length and other factors,^4 22–26^ we also considered a range of fixed values (RR=2–6) and found age-specific trends broadly consistent to overall BE prevalence results in CORI. Observed BE prevalence in white women undergoing screening was less precise in the CORI study data yet still coincided with our predictions for women (figure 3B).

In a sensitivity analysis, we also found these results to be robust to varying GERD prevalence in the model input for men and women in the population (see online supplemental material and online supplemental figure S1). When assuming smaller values of RR that lead to reduced BE prevalence in the GERD subpopulations for both sexes (see online supplemental material for details, online supplemental figure S2), the model still predicts that the majority of expected OACs (over 90%) develop in BE patients.

Finally, we previously found using this model that, for individuals born after 1940, the range of progression rates from BE-to-OAC was 0.10%–0.20% for men, and this was about twice as high as we found for women.^13^ These are plausibly low rates compared with current best estimates.^11 27^ Taken together, these secondary outcomes support the plausibility of our model’s predictions for numbers of OAC cases from BE annually.

The modelling results above imply that, even in the most conservative probability estimates, less than 10% of all annual OAC cases are unaccounted for beyond those expected to arise from BE. If there were a more significant alternate non-BE pathway than these numbers imply, then this model (which does not include a non-BE pathway) would have estimated either a much lower predicted population incidence of OAC than what was observed in SEER or shown greater inconsistencies with BE studies. In the latter case, the model would have estimated a greater prevalence of BE than what has been observed, and/or a greater rate of neoplastic progression among non-dysplastic BE than observed.

## Discussion

Based on the published epidemiology of BE and OAC, our analysis suggests that a major alternative non-BE pathway to OAC is an unlikely scenario. The existence of such an alternative pathway was suggested by a retrospective analysis of macroscopic reports of OAC specimens diagnosed without BE in two cohorts from the USA and UK by Sawas and colleagues; however, their study conclusions remain speculative due to some important limitations including (1) a lack of longitudinally followed cases to OAC from non-BE patient oesophageal tissue and (2) the plausibility that small BE segments were completely overtaken by malignant expansions and thus were unmeasurable at cancer diagnosis.^3^ Moreover, our result that BE is the main origin of OAC does not necessarily refute the existence of differing phenotypes for OAC—the finding that the presence of BE was associated with better survival could plausibly be explained by the theory that more aggressive cancers are likely to replace the precursor BE more readily than less aggressive cancers. The stochastic nature of our model allows for variation in progression across a population and we explored a wide range of parameter values for rates defining the stochastic process from birth to clinical OAC and reached similar results, but there is still ultimately some uncertainty.

Indeed, genetic and epigenetic analyses have also consistently shown BE and OAC to be very similar,^28–31^ and one study that sought genomic differences between adenocarcinomas with and without BE failed to reveal molecular differences between the two.^32^ Nonetheless, this is fortunate news that, with adequate uptake, screening for BE by upper endoscopy or minimally invasive non-endoscopic technologies^16 33^ could potentially identify and enrol all patients who are at risk for developing OAC into a surveillance programme.

Although the overall progression to OAC is low in patients diagnosed with BE, for those selected BE patients who have high grade dysplasia and/or early OAC detected during surveillance, effective treatment can save lives. In this way, our analysis reinforces the primary goal in BE screening for OAC prevention—that effective surveillance of the entire BE population could potentially prevent the majority of mortality caused by OAC in the general population. Further, by mathematically analysing the time-dependent nature of cumulative risk of BE in GERD patients, we can also use our multistage model framework to improve identification of at-risk populations by optimising the timing of initial screening recommended for BE in symptomatic GERD.^34^ Although current intensive ‘one-size-fits-all’ surveillance strategies^35–40^ would lead to high costs for those over-diagnosed BE screen cases and surveillance strategies clearly need to improve, we conclude that there is a strong rationale for screening for BE to reduce OAC mortality.

## Data Availability

All data relevant to the study are included in the article or uploaded as Supplementary material. All data used in our analysis are publicly available. CORI data can be accessed through application with ethical approval to NIDDK (https://niddkrepository.org/studies/cori/). All equations are provided either in Figures and Supplementary material or were previously published along with model parameters. Code to solve equations was developed in R (V.3.6.1). Computational scripts are available at: github.com/yosoykit/BEtoEAC_Results.
