# Supplementary material for: Computational modelling suggests that Barrett’s oesophagus may be the precursor of all oesophageal adenocarcinomas
Source: Gut. 2020 Nov 24;70(8):1435–40. doi: 10.1136/gutjnl-2020-321598 (PMC8292551; doi:10.1136/gutjnl-2020-321598)
Supplement: Supplementary data [file gutjnl-2020-321598supp001.pdf]

Supplementary Material

Model hazard function and expected number of esophageal adenocarcinoma (EAC) cases

We compared the model's predictions to the US population estimates for Barrett's esophagus (BE) and EAC quoted by Vaughan and Fitzgerald,[1] by combining age- and sex-specific model hazard rates with at-risk population estimates from US census data. [2] For expected EAC incidence, using the Markov model framework (see Figure 1) we can analytically compute the EAC hazard function h_{EAC} (see Curtius et al. for full derivation with age-specific GERD-dependent BE rates incorporated explicitly [3]) and estimate the expected number of newly diagnosed EAC cases by age and year separately for men and women, using population data for the at-risk population numbers.[2] This is computed as:

$$\Lambda_{i,j} = PY_{i,j} h_{EAC}(a_i, b_k),$$

where $PY_{i,j}$ is the number of person-years at-risk in period c_j of age a_i and birth cohort $b_k = c_j - a_i$. For the Age-Cohort model, the birth cohort specific hazard h_{EAC} (previously fit using person-year data for specified US populations directly from SEER [4]) can be written as $h_{EAC}(a_i, b_k) = h_{EAC}(t \mid t = a_i, b_k)$. For the main Results of incident EAC cases in $c_j = 2010$ and ages a_i between 40-90, this was computed separately for men and women and the 95% confidence interval for this estimate of summed total cases was computed by re-sampling Markov Chain Monte Carlo posterior distributions of birth year- and sex-specific model parameter estimates[3,4] for 100K bootstrap iterations. The equation below calculates the expected total number of EAC cases diagnosed in 2010, Λ_{2010}, which was equal to,

$$\sum_{i=40}^{90} \Lambda_{male_{i,2010}} + \Lambda_{female_{i,2010}} = \mathbf{9,970} \;\; [95\% \text{ CI}: \;\; 9,140 - 11,980]$$

For the analogous calculation using SEER incidence rates extracted from SEER*Explorer [5] for ages 40-90 in 2010 and census person-year data[2] we estimated 9,400 EAC cases total.

Sensitivity analysis: model input for gastroesophageal reflux disease (GERD) prevalence

The MSCE-EAC model *inputs* only include age- and sex- specific GERD prevalence and EAC incidence curves provided by SEER registry. Briefly, we previously developed a GERD model with GERD prevalence increasing in accordance with the data for age-specific GERD incidence (see Kong et al. 2014 for more detail [4]). The model also includes a parameter representing reversion of GERD symptoms, allowing us to fit age-adjusted GERD prevalence based on the US population between ages 40 to 85 to an approximate target of 20%, consistent with population-based studies of GERD prevalence. [6] This baseline input is provided in Figure S1A,B (solid blue line for males, and solid red line for females).

To determine the effect of varying GERD prevalence on the results for BE prevalence, we performed an additional sensitivity analysis to provide results based on explorations for a range of age-specific GERD prevalence, specifically between a 50% increase and decrease of values of the baseline function. Even with this range of GERD prevalence used as input (see Figure S1A,B), we found that the model's predictions for BE prevalence are robust to

changes in GERD prevalence with a narrow range of resulting BE prevalence produced as output for both men and women in the total population, which also still correspond well with CORI BE prevalence estimates (Figure S1C,D).

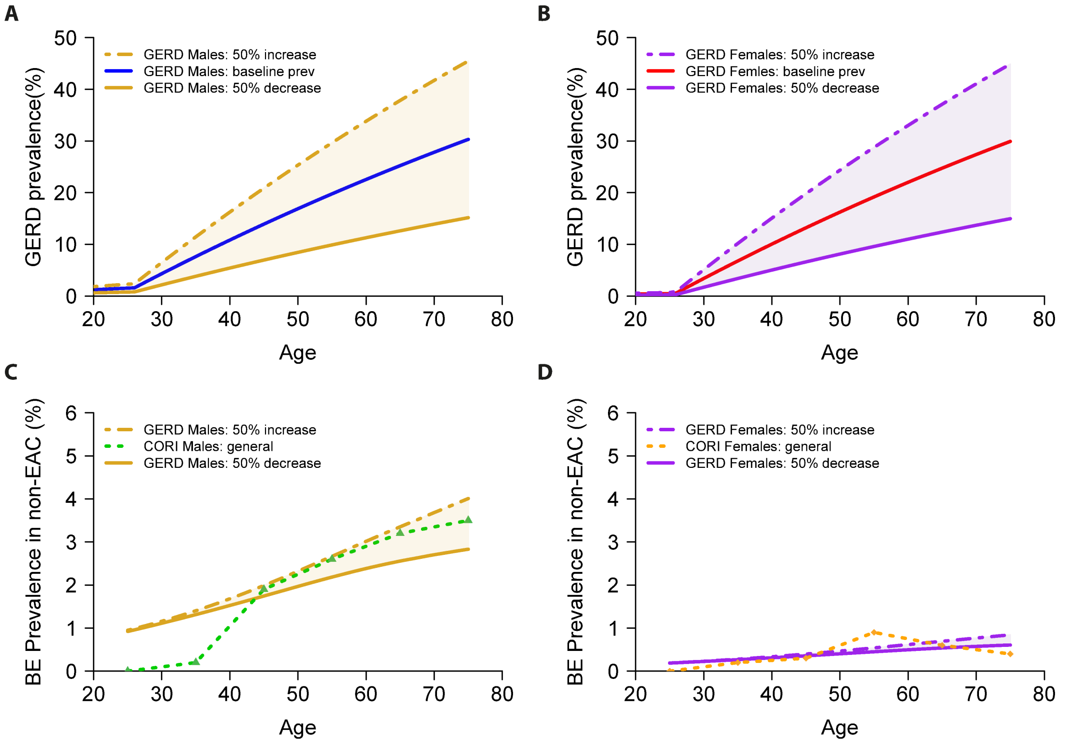

Figure S1: Sensitivity analysis for age- and sex- specific GERD prevalence model input in men (A) and women (B). With this input, corresponding estimates of BE prevalence in non-EAC population for main MSCE-EAC model results with total population considered are shown for men (C) and women (D) with RR=5. Shaded regions show general consistency with CORI data, and are similar to baseline Results in the main Text.

Sensitivity analysis: model input for relative risk (RR) of BE in GERD vs. non-GERD populations

We found that the model is very robust to changes in relative risk (RR) of BE among individuals with GERD symptoms compared with individuals without GERD symptoms. In the right panel of Figure S2, prevalence of BE in the GERD symptomatic population is displayed (governed by a GERD-specific BE rate $\nu_{GERD} = RR*\nu_0$ derived from the total model). When we assume RR to be equal to 4, which reduces the BE prevalence among individuals with GERD symptoms to 7.5% at age 55 and 8.7% at age 75 (right panel of Figure S2, grey dashed lines), the total number of EAC cases are estimated to be 9,904 [95% CI 9,037- 11,848]. Moreover, reducing the RR to 3 lowers the BE prevalence of men with GERD symptoms to 5.7% at age 55 and 6.6% at age 75 years (right panel of Figure S2, grey dotted lines) and the total number of EAC cases are estimated to be 9,843 [95% CI 8,930 – 11,719]. The reason the model is so robust in total number of predicted EACs is because the predictions for total population BE prevalence (Figure 3A; see left panel of Figure S2 below) are not very sensitive to changes in RR of GERD for BE (grey lines illustrate the narrow predicted range for BE prevalence), where the total model structure considers the age-dependency of the

prevalence of GERD symptoms, $p_{GERD}(t)$, in the time-dependent BE development rate, $\nu(t) = \nu_0[p_{GERD}(t)*RR+(1-p_{GERD}(t))]$, with baseline BE rate ν_0.

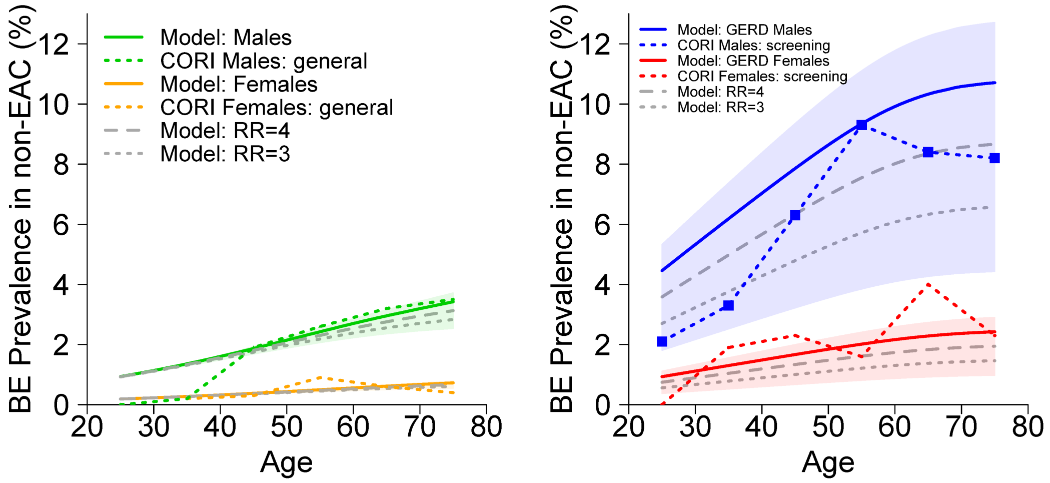

Figure S2: Sensitivity analysis for model predicted BE prevalence when lowering relative risk (RR) of BE in GERD versus non-GERD from the baseline value RR=5 (solid lines, see Figure 3 main Text) to RR=4 (dashed grey lines) and RR=3 (dotted grey lines). The total model predicted BE prevalence in men and women (left panel) is robust to changes in input for RR that consequently lower corresponding BE prevalence in GERD subpopulations (right panel).

Patient and Public Involvement

We did not directly include PPI in this study, but the SEER database used here is updated by an NCI committee that seeks quality improvement with patient/public feedback welcomed.

References

1. Vaughn TL, et al. *Nat Reviews Gastroenterol Hepatol* 2015;12:243-248.
2. United States Census Bureau. *Census of Population and Housing* [online], http://www.census.gov/prod/www/decennial.html (2015). Accessed Feb 2020.
3. Curtius K, et al. *PLoS Comput Biology* 2016 May 11;12(5):e1004919.
4. Kong CY, et al. *Cancer Epidemiol Biomarkers Prev* 2014;23(6):997-1006.
5. SEER*Explorer: An interactive website for SEER cancer statistics [online], https://seer.cancer.gov/explorer/ (2019).
6. El-Serag HB, et al. Update on the epidemiology of gastro-oesophageal reflux disease: a systematic review. *Gut* 2014;63(6):871-880.
